# Case report: A severe myositis mimicking bulbar palsy after administration of immune checkpoint inhibitors

**DOI:** 10.3389/fimmu.2025.1496427

**Published:** 2025-02-10

**Authors:** Philippe Rochigneux, Alexandre Bertucci, Elika Loir, Alexia Mattei, Danielle Robert, Michael Dassa, Brice Chanez, Mikael Ebbo, Lea Gaigne, Anne Sophie Chretien, Giovanni Corazza, Nicolas Schleinitz

**Affiliations:** ^1^ Medical Oncology Department, Paoli-Calmettes Institute, Marseille, France; ^2^ Team Immunity and Cancer, Centre de Recherche en Cancérologie de Marseille (CRCM), Inserm, U1068, Centre National de la Recherche Scientifique (CNRS), UMR7258, Paoli-Calmettes Institute, Aix-Marseille University, Marseille, France; ^3^ Otolaryngology Department, Assistance Publique Hôpitaux de Marseille, Aix-Marseille University, Marseille, France; ^4^ Radiology Department, Paoli-Calmettes Institute, Marseille, France; ^5^ Internal Medicine Department, Assistance Publique Hôpitaux de Marseille, Aix-Marseille University, Marseille, France; ^6^ Neurology Department, Assistance Publique Hôpitaux de Marseille, Aix-Marseille University, Marseille, France; ^7^ Referral Centre for Neuromuscular Diseases and Amyotrophic Lateral Sclerosis (ALS), Hôpital La Timone, Marseille, France

**Keywords:** immune-related myositis, bulbar palsy, immune checkpoint inhibitors, immune toxicity, ipilimumab/nivolumab

## Abstract

**Objectives:**

Immune Checkpoint Inhibitors (ICI) are nowadays a cornerstone of anti-cancer treatments. However, the wide spectrum of immune-related adverse events (irAEs) represents a challenge in the oncological practice. Our objective is to document rare complications of ICI to help the community of onco-immunologists.

**Methods:**

We reported the case of a severe myositis mimicking bulbar palsy treated in our Medical Oncology Department together with Internal Medicine Department. We present the clinical work-up (neurological exam, capillaroscopy) and the diagnostic tests (myositis specific and associated antibodies, nerve conduction study, electromyography) leading to this diagnosis. We also discussed the elimination of differential diagnoses (notably with normal MRI and cerebrospinal fluid analysis) and finally the clinical management of this severe irAE.

**Results:**

A 57 years woman presented multiple sub-diaphragmatic adenopathies related with an advanced melanoma of unknown primary. She started a treatment with Ipilimumab (Ipi, anti CTLA-4) and Nivolumab (Nivo, anti PD-1) and presented at day 10 a grade IV myositis mimicking bulbar palsy with dysphonia, dysarthria and aphagia. In a multidisciplinary setting, she was treated with IV corticosteroids (methylprednisolone 1 mg/kg started at day 10, with a progressive decrease until 1 mg of prednisone in March 2024), IV immunoglobulins started at day 18 (1.5 g/kg in 2 days, administered monthly, with a progressive decrease and a cessation in June 2022), enteral nutrition, speech therapy and physical therapy, with noticeable improvement. After 4 years of follow-up, and only one infusion of Ipi/Nivo, the melanoma is still in complete response.

**Conclusion:**

We report an ICI-induced severe myositis mimicking bulbar palsy after the administration of Ipi/Nivo. The diagnosis and clinical care management of this rare complication requires a multi-disciplinary work-up.

## 1 Case presentation

**Figure 1:**
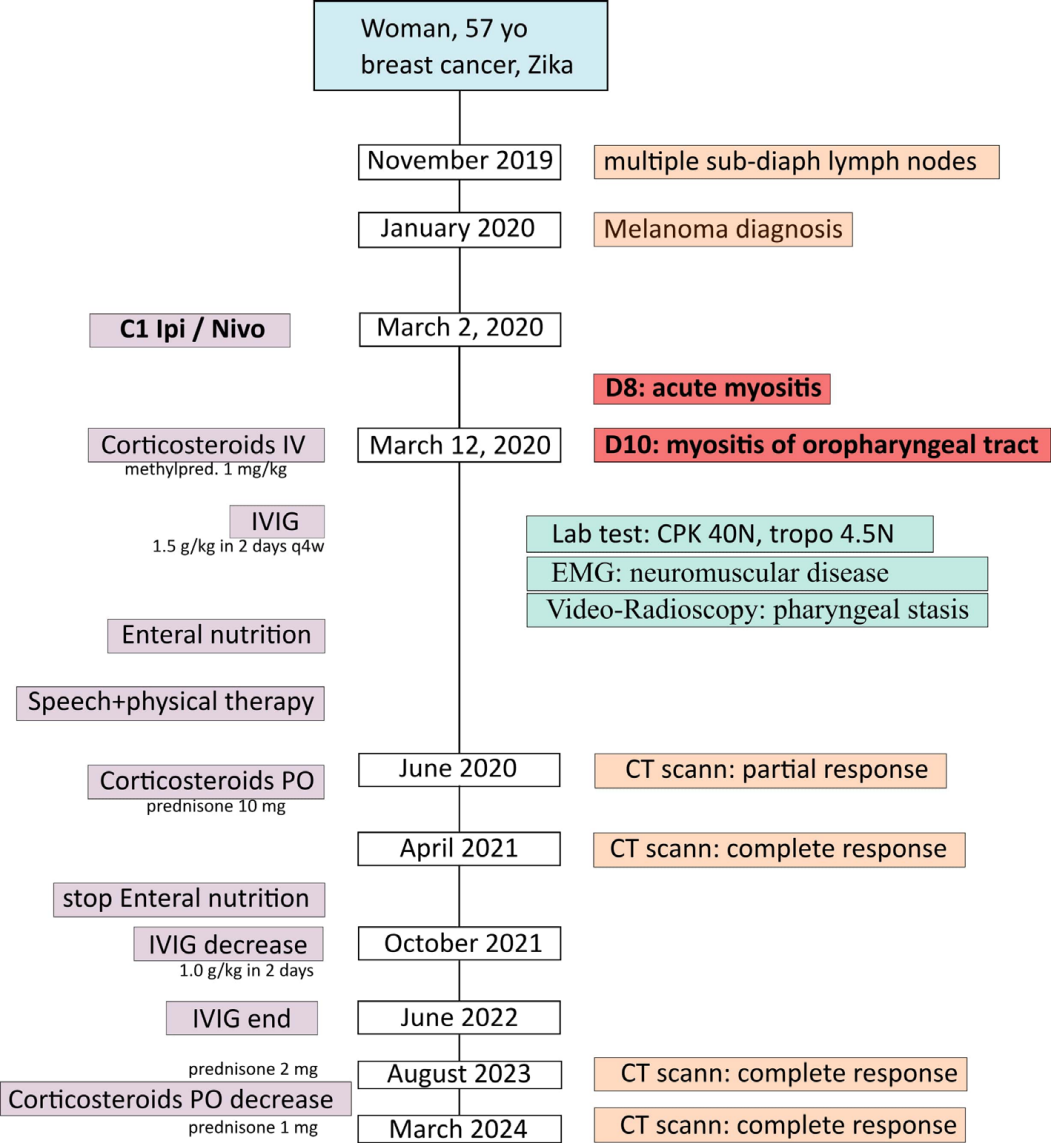
Patient’s timeline.

A 57 year-old woman, ECOG 0, with a medical history of locoregional, Hormone Receptor positive (HR^+^) breast cancer (pT2N2M0: treated by surgery, adjuvant chemotherapy by anthracyclines/cyclophosphamide (EC) and paclitaxel, radiotherapy and anti-aromatase), asthma, Zika and Chikungunya infection, presented in November 2019 a discomfort and burning in the left side pubic area (**Figure 1**). Then, she noticed a left inguinal adenopathy, increasing in size. Mid-January 2020, she consulted in internal medicine at a general hospital, where lab tests were normal (notably for HIV, plasma protein electrophoresis). An abdomino-pelvic CT scan on January 10, 2020 revealed several adenopathies: left inguinal (17 mm), left external iliac (34 mm), left internal iliac (41 mm) and left latero-aortic (28 mm). The surgical lymphadenectomy revealed a malignant melanoma with sarcomatoid dedifferentiation (PS100^-^, MelanA^-^, BRAF^-^), with NRAS mutation on Exon 3 (p.Gln61Leu).

The patient was transferred to our Cancer Center (Institut Paoli-Calmettes, UNICANCER Marseille, France). Physical examination and PET-CT did not reveal the primary site of this melanoma and symptoms quickly worsened (oedema of the left lower limb and the pubis, ilio-femoral venous thrombosis). Tumor board validated a combined immunotherapy with ipilimumab 3 mg/kg (anti CTLA-4) and nivolumab 1 mg/kg (anti PD-1) with a first infusion in March 2, 2020.

Two days after immunotherapy, she presented asthenia and severe headaches. Then, on day 8, she noticed the onset of bilateral oedema in the wrists, associated with slight pain in the thumb, rising to the elbow, associated with hyperthermia. On day 10, she also described swallowing difficulties with a sensation of dysphonia. She was admitted in the emergency department: swallowing disorders increased until day 12, with dysphagia to solids preventing any feeding, together with a dysphonia and a sensation of para-cervical stricture. Lab tests revealed hepatic cytolysis (from 10N to 15N), with moderated cholestasis (GGT 1.5N) and elevated CPK (4574 to 5898 U/L, 40N with ULN: 145 U/L) and troponin (53 ng/L, 4.5N with ULN: 11.6).

We started IV corticosteroid (1 mg/kg methylprednisolone) and the patient was transferred to the Internal Medicine Department. Hence, the clinical exam revealed a bilateral deficit in digital extension muscles, together with hypoesthesia and dysesthesia of the right inferior limb. Initially, no motor deficit was observed in the lower inferior limbs, but a motor deficit appeared 4 months after the C1D1 (July 2020) in the right foot elevator muscles (4/5) and in the right ilio-psoas muscle (4/5). The 8-finger capillaroscopy revealed “branched capillaries”, signing organic microangiopathy, as seen in myositis (1). Testing for myositis specific and associated antibodies remained negative, as anti-RAC and anti-MusK antibodies (anti-striational antibodies not tested) and we did not conduct muscle biopsy.

Clinical examination and nerve conduction study (NCS) were quickly performed by a neurologist. There was a clinical deficit in ulnar (4/5) and median innervated muscles (2/5) in addition with previously described deficits. NCS assessed tibial and common fibular nerves on lower limbs, and median and ulnar nerves on upper limbs. It showed decreased SNAP and CMAP in the right fibular nerve, left ulnar nerve and both median nerves. Conduction velocity and F-waves latency were normal in all nerves. Electromyography (EMG) reported a myogenic damage of the glossus muscle whereas right dorsal interosseous, right vastus lateralis and right tibialis anterior were normal. Radial/median nerves innervated muscles and iliopsoas were not assessed. Repetitive nerve stimulation was normal in all studied muscles (trapezius, anconeus, tibialis anterior, abductor digiti quinti, and mentalis on both sides; and brevis abductor pollicis on right side).

Importantly, electrocardiogram and echocardiography were normal, as well as brain MRI. The cerebrospinal fluid analysis was normal, without pathological cell in cytology, 2.4/mm^3^ white blood cells, 3.1/mm^3^ red blood cells, protein 0.43 g/l, glucose 4.79 mmol/l.

The respiratory function tests revealed normal spirometry and lung volumes, normal lung diffusion capacity (77% of theoretical value), but a decrease in maximal inspiratory pressure (lowered to 37cmH20; 51% of theoretical value).

**Figure 2:**
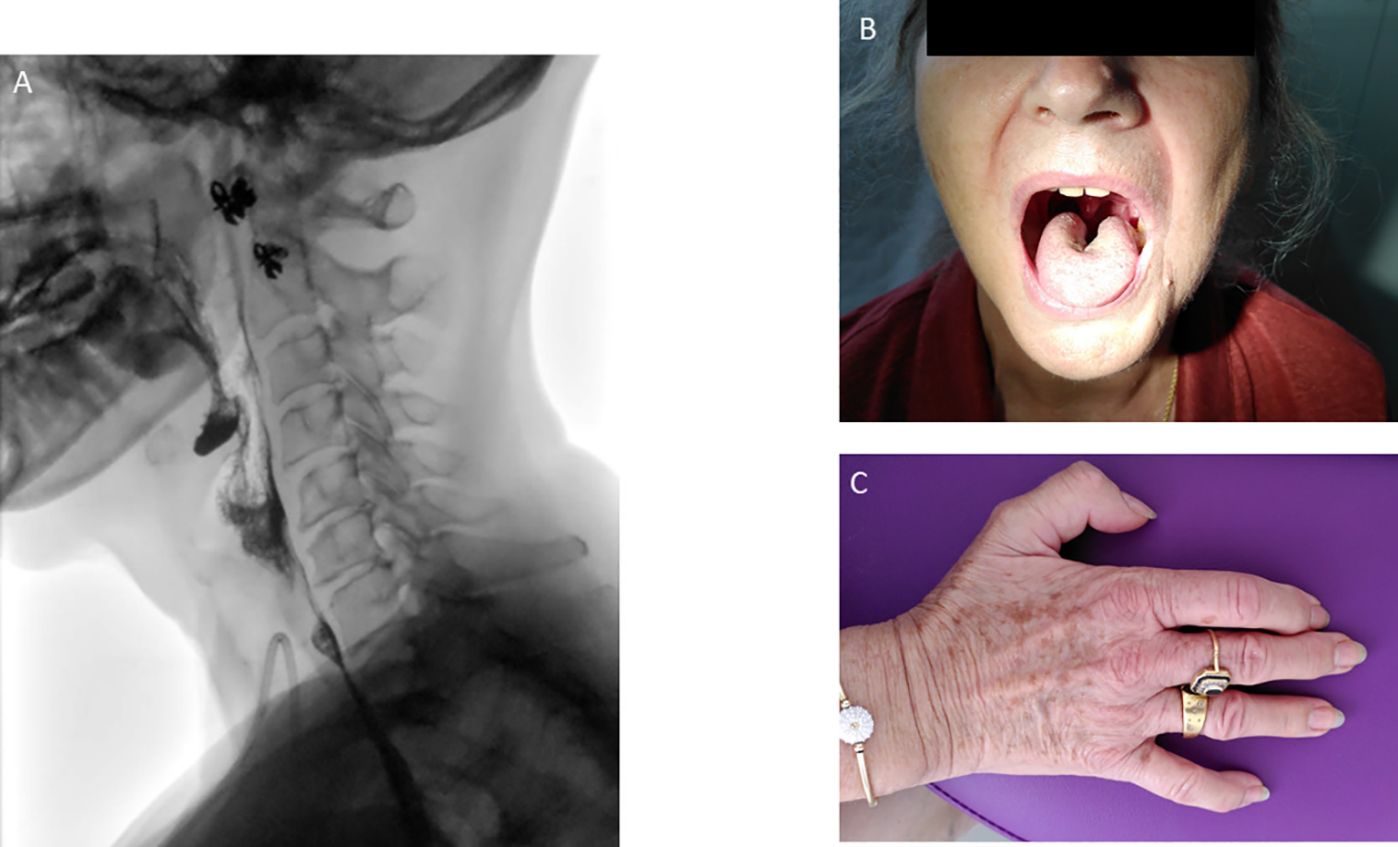
Modified barium swallow leading to oral, vallecular and hypopharyngeal residue **(A)**. Central lingual atrophy **(B)** plus hypotrophy of the dorsal interosseous muscles **(C)** observed during the follow-up.

Fiberoptic endoscopic evaluation of swallowing and videofluoroscopy showed a diffuse muscle impairment with a decreased base-of-tongue retraction, a decreased pharyngeal squeeze leading to oral, vallecular and hypopharyngeal residue (**Figure 2**; **Supplementary Video 1**), but without penetration or aspiration. During the follow-up (October 2020), the patient developed a lingual atrophy (**Figure 2B**).

Finally, we retained the diagnosis of grade IV ICI-induced myositis and mononeuritis multiplex with distal weakness of the superior limbs and dysphonia, dysarthria and aphagia (mimicking bulbar palsy).

## 2 Treatment

The patient was treated with IV corticosteroids, methylprednisolone 1 mg/kg started at day 10 (March12, 2020) and infusion of IV immunoglobulins (1.5 g/kg in 2 days) every 4 weeks started at day 18 (March 20, 2020). After concertation between oncologists and internists, Ipi/Nivo combination was stopped after this single infusion. Concerning supportive care, parenteral nutrition was initially started, but in May 2020 a gastrostomy allowed enteral nutrition. She quickly started physical therapy and speech therapy.

The clinical evolution revealed a slow improvement of the neuro-muscular symptoms. Corticosteroids were switched *per os* and the posology was progressively decreased until prednisone 10mg in July 2020. At this time, the patient could walk 15 min and climb stairs with help, had a slight improvement in dysphagia (yoghurt and mixed meals) but dysphonia persisted. In November 2020, as the patient could climb stairs with help and had a phonation improvement, oral prednisone was decreased to 5 mg, to 2 mg in August 2023 and finally 1 mg in March 2024. The IV immunoglobulins were also decreased (1 g/kg every 2 months in October 2021) and were finally stopped in June 2022. Dysphagia and dysarthria were the main persistent symptoms. Patient had close follow-up with otolaryngologists, nutritionists and speech therapists. In October 2021, the enteral nutrition was stopped and the gastrostomy was removed in January 2023. During the follow-up, she also suffered from central lingual atrophy in October 2020 (**Figure 2B**) plus hypotrophy of the dorsal interosseous muscles (**Figure 2C**). Biologically, CPK levels decreased to 1227 U/l in May 2020 and normalized in July 2020.

**Figure 3:**
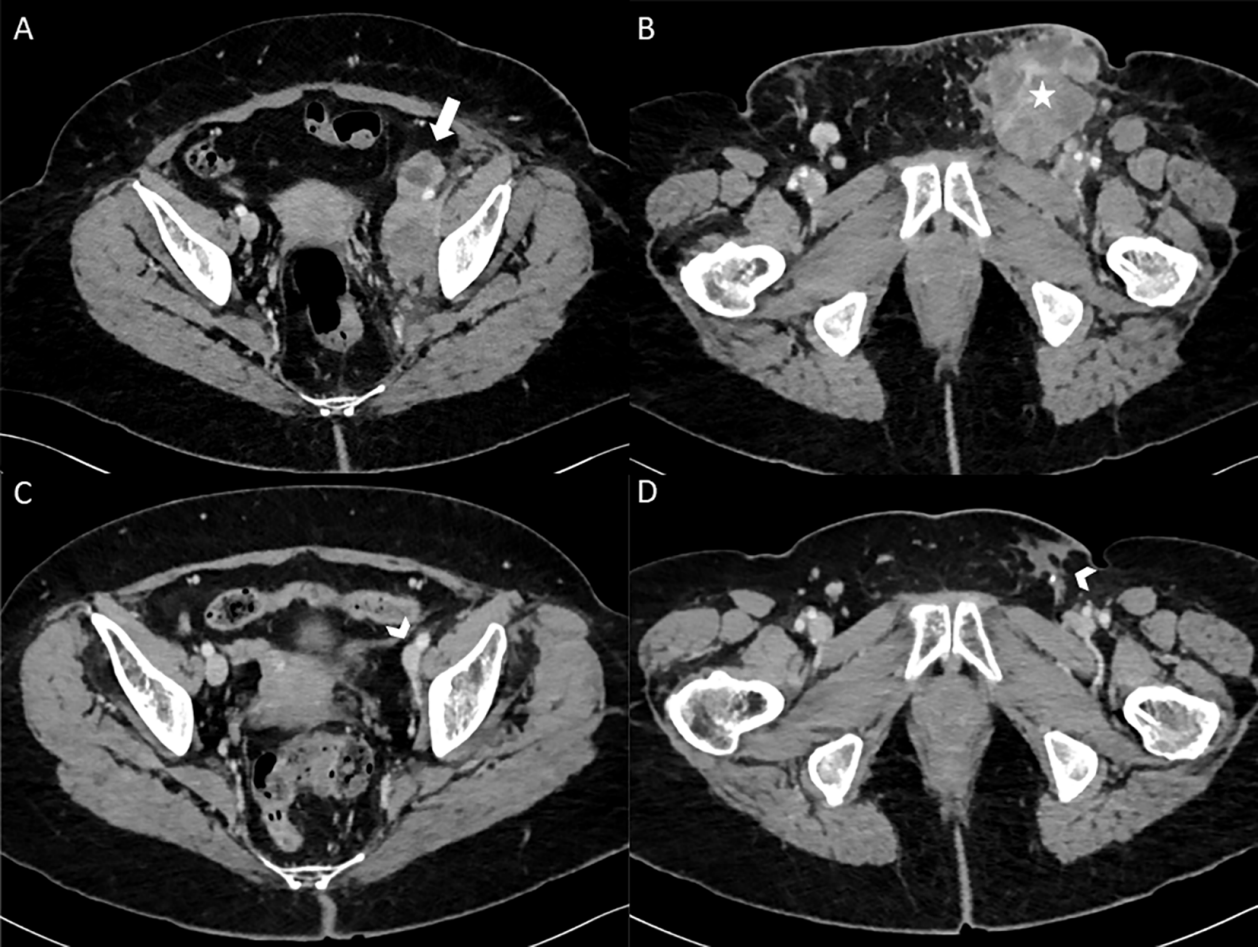
Enhanced CT scanner images (axial view) at baseline in February 2020 **(A, B)** showing left external and internal iliac lymphadenopathy (thick white arrow) and left inguinal lymphadenopathy (white star). After 1 Ipilimumab/Nivolumab infusion with 3.5 years of follow-up in August 2023 **(C, D)**, enhanced CT scanner (axial view) only report lymph nodes remodeling signs (arrowheads) without any sign of active tumors.

In an oncological perspective, the first CT-scans of June 2020, 3 months after the unique Ipi/Nivo infusion showed a clinically meaningful partial response (>50%) in left iliac and latero-aortic lymph nodes. The following CT-scans confirmed a gradual decrease of left inguinal adenopathies, until 9 mm of small axis in April 2021. Since June 2022, the patient CT scanners only report lymph nodes remodeling signs without any sign of active tumors (**Figure 3**). Altogether, the patient displays of complete tumor response after a single Ipi/Nivo infusion, without any other oncological treatments, persisting after more than four years of follow-up.

## 3 Differential diagnosis

In this patient, we retained the diagnosis of peripheral neuropathy (mononeuritis multiplex) and grade IV ICI-induced myositis mimicking bulbar palsy.

On the first clinical exam, we noticed a diffuse lingual and pharyngeal muscle deficit (with dysarthria, dysphagia), without fasciculation and amyotrophy. According to this clinical presentation, central nervous system lesion leading to pseudo-bulbar palsy need to be seeked and excluded. *Stricto sensu*, pseudo-bulbar palsy corresponds to a lesion of upper motor neurons, representing a clinical constellation characterized by dysarthria, dysphagia, facial and tongue weakness, and emotional lability. This syndrome might be due to traumatic brain injury, neoplasm, vascular lesions, metabolic abnormality, or neurological disease. Notably, in the literature, acute pseudo-bulbar palsy is often related to stroke with magnetic resonance imaging showing an interruption of the corticonuclear pathways (2, 3). In our case, we did not notice emotional lability, spastic voice, spasmodic laughing or crying, and brain MRI was normal, eliminating acute pseudo-bulbar palsy.

In an oncological perspective, excluding brain or leptomeningeal metastases is also necessary. However, although described in the literature, acute pseudo-bulbar or supra-bulbar palsy is rarely related to neoplastic CNS involvement (4). To rule out leptomeningeal metastases, lumbar puncture is crucial, with a sensitivity around 50-60% if isolated and 80% if repeated (5).

Moreover, in the context of neurological symptoms during ICI treatments, one should also exclude neurological AES involving the central nervous system, causing encephalitis, meningitis, or myelitis (6). Notably, ICI-induced encephalitis is feared by medical oncologists because of its high mortality rate (around 25%) (7). In ICI-induced encephalitis (a differential diagnosis of this case), brain MRI typically displays bilateral brain abnormalities in FLAIR and lumbar puncture shows pleocytosis in the cerebrospinal fluid. If there is no clinical evidence of CNS involvement, clinical examination, electrophysiological study and biological exams should be performed to diagnose neuromuscular disorders that mimic pseudo-bulbar syndrome. Myasthenia gravis, a condition affecting the neuromuscular junction frequent in thoracic malignancies and reported with ICI, must be sought, as inflammatory neuropathies such as pharyngeal-cervical-brachial syndrome, Guillain-Barre’s variant affecting cranial nerves (8, 9).

Finally, in a neurological perspective, the patient displayed a non-length-dependent asymmetric sensorimotor deficit. Considering the time separating her previous chemotherapies from this new event, and the clinical and electrophysiological features, we have retained the diagnosis of mononeuritis multiplex. Interestingly, two recent studies (a case report and a case series of three cases) report this diagnosis during ICI treatment (10, 11). Consequently, clinicians should be aware of this complication in the context of ICI.

## 4 Discussion

In the field of clinical immunotherapy, several lessons can be learned from this case. First, ICI-induced myositis can affect every muscle, including oropharyngeal ones. ICI-induced myositis occurs in 1% of patients treated with anti PD-1 antibodies, but myalgia was reported in around 5% of patients in clinical studies investigating anti PD-1 monotherapy (12). Classically, ICI-induced myositis is more frequent in limb-girdle, axial, oculomotor or cardiac muscles (13, 14). Here, the occurrence of ir-myositis of oropharyngeal muscle is unusual and might constitute a diagnostic pitfall (15). In the literature, bulbar myopathy is described (16), but remained a rare entity, with a severe prognosis (17). Oncologists must be aware of this oropharyngeal ir-myositis location, which may result in severe loco-regional complications. Indeed, the onset of ir-myositis is often acute, which may lead to unexpected respiratory insufficiency (17).

Consequently, the 2^nd^ lesson is that the clinical management of oropharyngeal ir-myositis is urgent (18). Indeed, next to respiratory insufficiency, myocarditis is associated in around 30% of myositis (12). Hence, differential diagnoses and associated diagnoses must be quickly eliminated to start corticosteroids as soon as possible (19). In that perspective, it seems highly important for oncologists involved in immunotherapy to settle a close collaboration with neurologists and internists as quick clinical evaluation, quick imaging and lab tests are essential to eliminate differential diagnoses. Indeed, especially if combined with myocarditis, severe myositis must lead to rapid medical treatments to prevent fatal complications. First, high-dose intravenous methylprednisolone must be quickly initiated. But in glucocorticoid-refractory patients, secondary immunosuppressants such as infliximab, rituximab, and mycophenolate mofetil, abatacept must be discussed with internists, cardiologists, neurologists and intensive care physicians (13, 20).

This essential collaboration in diagnosis work-up and initiation of specialized immunosuppressive treatments can take various forms, such as external consultants, immuno-toxicity meetings, mailing lists, messaging applications (21). Building a strong network to deal with rare and life-threatening irAEs is one of the new challenge of clinical immuno-oncology.

Thirdly, side effects of severe myositis may result in permanent sequelae. In a case series of 38 patients (12), half of the cases (50%) were completely resolved, whereas the rest was either ongoing (26%) or had sequelae (16%). Hence, the functional rehabilitation of ir-myositis is crucial. Particularly, oropharyngeal ir-myositis requires multiple healthcare professionals to improve patient’s quality of life, notably otolaryngologists, speech therapists, physiotherapists and nutritionists. Fortunately, in our patient, the functional rehabilitation led to significant improvements, but required a strong implication of the team and multiples efforts from the patient.

## Data Availability

The raw data supporting the conclusions of this article will be made available by the authors, without undue reservation.
